# Inhibition of C5a-C5aR1 axis suppresses tumour progression by enhancing antitumour immunity and chemotherapeutic effect in pancreatic ductal adenocarcinoma

**DOI:** 10.1038/s41416-025-03185-0

**Published:** 2025-10-03

**Authors:** Ryotaro Eto, Shigetsugu Takano, Daren Zhou, Kensuke Suzuki, Tsukasa Takayashiki, Daisuke Suzuki, Nozomu Sakai, Masayuki Ohtsuka

**Affiliations:** Department of General Surgery, Chiba University, Graduate School of Medicine, Chiba, Japan

**Keywords:** Cancer microenvironment, Cancer immunotherapy

## Abstract

**Background:**

Complement factors regulate tumour immunity in the tumour microenvironment (TME). We investigated the functions of complement 5a (C5a) and its receptors, C5aR1 and C5aR2, in forming the C5a-C5aR1 and C5a-C5aR2 signaling axes in the immune TME of pancreatic ductal adenocarcinoma (PDAC).

**Methods:**

C5a, C5aR1 and C5aR2 were assessed in cancer cell cytoplasmic (c-) and stromal (s-) expressions in resected PDAC tissues. In vitro assays were conducted to examine endogenous C5aR1 functions in PDAC cells, and orthotopic transplantation was performed in a preclinical study.

**Results:**

In immunohistochemistry, High C5a-C5aR1 c-axis was correlated with poor prognosis and High C5a-C5aR1 s-axis was associated with a decrease in CD8^+^ T cells and an increase in CD11b^+^ MDSCs. C5aR1 knockdown and CCX168, the specific C5aR1 inhibitor, impaired proliferation and the activation of the PI3K/mTOR pathway, and enhanced gemcitabine sensitivity by increasing apoptosis. The combination of CCX168 and gemcitabine/nab-paclitaxel demonstrated a significant reduction in tumour volume. The number of CD8^+^ T cells was significantly increased in CCX168-treated groups, whereas CCX168 treatment resulted in a decrease in MDSCs.

**Conclusions:**

The C5a-C5aR1 axis may exert a tumour-promoting effect on the TME in PDAC. CCX168 appears to modulate antitumour immunity, thereby warranting future complement-based immunomodulation therapies for PDAC.

## Background

Among all malignancies, pancreatic ductal adenocarcinoma (PDAC) exhibits poor outcomes, with an overall 5-year survival rate of approximately 13% [1, 2]. To enhance the prognosis, extensive research is underway to explore new avenues to overcome resistance to conventional treatments across various domains, such as surgery, chemotherapy, radiotherapy, and immunotherapy [3]. Resistance to treatment is largely attributed to the tumour microenvironment (TME), which is a complex system composed of the extracellular matrix, immune cells, chemokines, and secreted proteins that influence the mechanisms underlying tumour progression.

Recently, the complement system has been recognised as either a suppressor or promoter of cancer progression, depending on the specific organ and the type of complement involved [4, 5]. Accumulating evidence has implicated the functional roles of tumour progression in the TME [6–8]; thus, the complement system may contain attractive targets for novel therapies. However, the impact of interactions between complement factors and tumour immunity on the mechanisms of tumour progression has not been fully addressed.

We previously demonstrated that complement 4b-binding protein alpha chain (C4BPA), a component of the complement pathway, influences the TME of PDAC by enhancing antitumour immunity through the recruitment of CD8^+^ T cells to the tumour periphery. In an in vivo study, the C4BPA peptide exhibited antitumour effects against PDAC when used in combination with existing chemotherapy and immune checkpoint blockers (ICBs) [9, 10]. We have also reported that complement factor B (CFB) secreted by PDAC cells regulates senescence, leading to the promotion of tumour progression within the TME of PDAC [11]. Thus, we hypothesised that understanding the mechanisms of the complement cascade in the TME, especially the orchestration of complement factors and immune TME, could lead to the discovery of novel strategies for PDAC treatment.

In this study, we focused on complement 5a (C5a) and its receptors, C5a receptor 1 (C5aR1) and C5a receptor 2 (C5aR2), to form a signalling axis located downstream of C4BPA and CFB in the complement cascade. We aimed to investigate the correlation between C5a-C5aR1/2 and the immune TME in resected PDAC tissues. Furthermore, we conducted a preclinical study to investigate the potential of the C5a-C5aR1 axis as a novel therapeutic target for PDAC. Our experimental findings demonstrate that the C5a-C5aR1 axis predominantly plays a promotive role in PDAC progression in progressive tumour dynamics; thus, inhibition of C5aR1 could be a potential therapeutic target for PDAC.

## Methods

### Human clinical specimens

To investigate the expression of the C5a-C5aR1/2 axes and their correlation with clinical prognosis, tumour tissues were extracted from 130 PDAC patients who were performed pancreatectomy without any preoperative treatments at our institution from January 2014 to December 2018. This study was approved by the Ethics Board of Chiba University (#2958), and informed consent was obtained from all patients for the use of their individual specimens.

### Immunohistochemistry (IHC) and immunofluorescence (IF) staining

IHC was performed according to a standardised protocol. Paraffin blocks prepared at our institution were sectioned into 4-µm thick slices to create unstained preparations. Following deparaffinization, antigen retrieval was performed using a citrate buffer (pH 9) and autoclaving for 120 min. After blocking with methanol, subsequent blocking with bovine serum albumin was performed for 40 min, and primary antibodies were applied and allowed to incubate overnight at 4 °C. Secondary antibodies were then added and incubated at room temperature for approximately 60 min.

IHC was performed using haematoxylin, followed by fixation through exposure to ethanol and xylene and cover glass mounting with Marinol. For IF staining, cover glass mounting was performed using 4′,6-diamidino-2-phenylindole for nuclear staining. The detailed information of C5a, C5/C5b, C5aR1, and C5aR2 antibodies was indicated as follows;

Human C5a mouse monoclonal antibody (D2): cat. no. MA5-43875, Company: Thermo Fisher Scientific Japan, Degree of dilution for IHC and IF: 1:100;

Human C5/C5b monoclonal antibody: cat. no. HA255621, Company: ABclonal, Degree of dilution for IHC: 1:100;

Human C5aR1 rabbit polyclonal antibody: cat. no. A1900, Company: ABclonal Hubei China, Cross-reactivity: mouse, rat, Degree of dilution for IHC and IF: 1:150;

Human C5aR2 rabbit polyclonal antibody: cat. no. HPA016629, Company: Merck Ltd./Sigma-Aldrich Japan G.K., Degree of dilution for IHC and IF: 1:75.

The expression patterns of C5a, C5aR1, and C5aR2 were assessed with internal positivity defined in the islets of Langerhans within each sample. High expression was categorised as levels equal to or greater than those observed in the cancer cell cytoplasm or stromal cells, whereas low expression was defined as levels lower than those in the cancer cell cytoplasm or stroma. Additionally, IHC for CD8 and CD11b was used to quantify CD8^+^ T cells and CD11b^+^ myeloid-derived suppressor cells (MDSCs), respectively, in the vicinity of the PDAC. The mean number of immune cells was counted in three different fields of view at a magnification of ×400. The IHC scores were independently evaluated by three investigators.

### Human and murine cell lines

Human pancreatic ductal epithelial (HPDE) cells and PDAC cell lines (BxPC-3, PANC-1, MIA PaCa-2, Capan-2, Capan-1, AsPC-1, CFPAC-1, and Hs766T) were obtained from the American Type Culture Collection. Murine PDAC cell lines derived from Pdx1-Cre; LSL-Kras^G12D/+^; Trp53^R172H/+^; Rosa26^YFP/YFP^ (KPCY) mice, specifically KPCY2838 and KPCY6419, were provided by Dr. Andrew D. Rhim at the University of Texas, MD Anderson Cancer Center. MIA PaCa-2, PANC-1, and Hs766T cell lines were maintained in Dulbecco’s modified Eagle’s medium (DMEM; Sigma-Aldrich) supplemented with 10% fetal bovine serum (FBS). Capan-2 cells were cultured in McCoy’s 5A medium (Cytiva, Issaquah, WA) supplemented with 10% FBS. BxPC-3 and AsPC-1 cells were cultured in RPMI-1640 medium (Thermo Fisher Scientific, Waltham, MA) with 10% FBS. CFPAC-1 cells were maintained in Iscove’s modified Dulbecco’s medium (Thermo Fisher Scientific) supplemented with 10% FBS and antibiotics, whereas Capan-1 cells were cultured in DMEM supplemented with 10% FBS.

### Small interfering RNA (siRNA) and short hairpin RNA (shRNA) transfection

C5aR1-specific siRNAs were utilised for gene silencing, specifically C5aR1siRNA1 (Cat. SI00027412, Lot. 40202992; target sequence: CCCAGGAGACCAGAACATGAA; QIAGEN) and C5aR1siRNA2 (Cat. SI00027419, Lot. 40202991; target sequence: CCGGAACGTGTTGACTGAAGA; QIAGEN), along with a negative control siRNA (QIAGEN). MIA PaCa-2 and BxPC-3 cells were transfected with these two siRNAs at a concentration of 5 nmol/L using Lipofectamine RNAiMAX reagent (Invitrogen, Carlsbad, CA). Additionally, lentiviral transfections with two C5aR1 shRNAs were conducted in KPCY2838 and KPCY6419 cells. Negative control lentiviral vector (LVC; cat. VB010000-0009mxc) and C5aR1shRNA-1 (Cat. VB900035-7555scb, Target sequence: GCACACTGTATGTGGTATTAA) and C5aR1shRNA-2 (Cat. VB900035-7560bxy, Target sequence: CGTGTACCGGGAGGCATATAA) was obtained from VectorBuilder. Following lentiviral infection, KPCY cells were selected using 5 µg/mL puromycin. To confirm the effectiveness of the knockdown of C5aR1, western blotting was performed, with β-actin utilised as a normalisation control for evaluation.

### Western blotting

Total protein was extracted from cultured cells using radioimmunoprecipitation buffer (Sigma-Aldrich) and stored at −80 °C. Twenty micrograms of protein were loaded onto 5–12.5% XV PANTERA gels (DRC, Tama, Tokyo, Japan) and transferred to polyvinylidene fluoride membranes. Nonspecific protein binding was blocked using 2% skim milk and incubated at room temperature for 60 min. Following blocking, the membranes were incubated overnight at 4 °C with primary antibodies targeting C5a, C5aR1, C5aR2, E-cadherin, Vimentin, Snail, total PI3K (t-PI3K), phosphorylated PI3K (p-PI3K), total AKT (t-AKT), phosphorylated AKT (p-AKT), total mTOR (t-mTOR), phosphorylated mTOR (p-mTOR), and anti-β-actin (Cell Signaling Technology, Danvers, MA). Membranes were then incubated with secondary antibodies at room temperature for 60 min. After exposure to the detection reagents, the membranes were analysed using an LAS-4000 UV image analyser (Fujifilm, Tokyo, Japan). Western blot results were quantified by densitometric analysis and normalised against β-actin using ImageJ software.

### Quantitative RT-PCR

RNA was extracted from cell lines (MIA PaCa-2, BxPC-3) using the RNeasy Mini Kit (Qiagen). RNA was reverse transcribed to cDNA using the SuperScript VILO cDNA Synthesis Kit and Master Mix (Life Technologies). Gene expression was quantified using the SYBR Green method with TB Green Fast qPCR Mix (TaKaRa Bio Inc., Shiga, Japan) and human C5 primers (Forward: GCAAATGCAGATGACTCCCAAG, Reverse: CGCAGGCTCCATCGTAACAA; TaKaRa Bio Inc). The ΔCT value was calculated. The CT value of C5 was normalised to β-actin and error bars represent the mean ± standard error of the mean.

### Enzyme-linked immunosorbent assay (ELISA)

We cultured cell lines (MIA PaCa-2, BxPC-3) with serum-free medium and subsequently extracted the supernatant and cell lysates. An anti-C5a antibody was dispensed into a plate and incubated at a concentration of 0.5 mg/well for 1 day at 4 °C for coating (Human Complement C5a ELISA Kit, cat. no. #BMS2088, Thermo Fisher). After immobilisation, the cell culture supernatants were applied to the plate and then reacted for 1 h at room temperature. The detect antibody was incubated for 30 min at room temperature. The absorbance of the solution was measured at 450 nm and error bars represent the mean ± standard error of the mean.

### Proliferation assay

At 24 h post-transfection with C5aR1siRNAs, MIA PaCa-2 and BxPC-3 cells were detached and seeded at a density of 2 × 10^3^ cells per well in 96-well plates for proliferation assays. Additionally, C5aR1shRNA-transfected KPCY2838 and KPCY6419 cells were seeded in 96-well plates at the same density for the proliferation assays. For drug-based assays, 2 × 10^3^ cells were seeded in a 96-well plate and treated with gemcitabine (Selleck, Cat. No. S1714) (20 ng/mL) and CCX168 (Selleck, Cat. No. S0474) (171.9 ng/mL) and incubated in a 5% CO_2_ atmosphere at 37 °C. Cell proliferation was assessed using the Cell Count Reagent SF (Nacalai Tesque) according to the manufacturer’s protocol. Cell viability was determined by measuring the absorbance at 450 nm using a plate reader (Bio-Rad Laboratories) at 24, 48, 72, and 96 h after cell seeding. Each experiment was independently conducted thrice, resulting in three replicates. Statistical significance was defined as *p* < 0.05 (Mann–Whitney–Wilcoxon test), and error bars represent the mean ± standard error of the mean.

### Apoptosis assay

Using the Annexin V-FITC Apoptosis Detection Kit (Nacalai Tesque, Kyoto, Japan), 1 × 10^6^ cells were suspended in 100 µL of phosphate-buffered saline (PBS) and analysed utilising the Cell Counting Kit-8 alongside the Annexin V-FITC/propidium iodide (PI) double staining method. Apoptotic cells were identified by flow cytometry, and a minimum of 1 × 10^4^ cells per sample was analysed.

### Pancreas orthotopic transplantation mouse model

In the pancreatic orthotopic transplantation mouse model, 8–10-week-old female C57BL/6JJcl mice (CLEA Japan, Tokyo, Japan) were injected with 1 × 10^6^ KPCY2838 cells suspended in 25 µL of DMEM supplemented with 10% FBS, into the capsule of the pancreatic tail under inhalation anaesthesia using sevoflurane, thereby generating orthotopic transplantation model mice [12]. Following the study protocol, mice were euthanized on day 19 after orthotopic transplantation, and the primary pancreatic tumour, attached spleen, and entire liver were excised. The mean tumour volume was measured to determine the baseline tumour volume. For histological analysis, the excised pancreas and liver were fixed in 4% paraformaldehyde for 18 h, transferred to 100% ethanol, and embedded in paraffin. The volume of the primary tumour was measured using a vernier caliper and calculated with the following formula: *π*/6 × (*L* × *W* × *W*), where “*L*” represents the longest dimension of the tumour and “*W*” denotes the shortest dimension [13]. Primary tumours were analysed by microscopic examination using haematoxylin and eosin (HE) and IHC staining.

### Murine in vivo antibodies and drug preparation

Mice were treated with pharmaceutical grade Gem (Selleck, Cat. No. S1714) at a dosage of 120 mg/kg and nab-Paclitaxel (Selleck, Cat. No. E1068) at 30 mg/kg dissolved and suspended in corn oil. A combination of gemcitabine and nab-paclitaxel (GnP) was administered intraperitoneally. Pembrolizumab (Selleck, Cat. No. A2005), an anti-PD-1 antibody, was administered intraperitoneally at 5 mg/kg along with ipilimumab (Selleck, Cat. No. A2001) at 1 mg/kg, collectively referred to as ICBs. These antibodies were dissolved in PBS and confirmed to be endotoxin-free. CCX168 (30 mg/kg) was administered by oral gavage and suspended in corn oil. The mice were sacrificed 19 days after cell injection and the mean tumour volume was measured using vernier calipers. Primary tumours were analysed microscopically via HE and IHC staining. CD8, CD11b and cleaved caspase-3 immunostaining was used to quantify CD8^+^ T cells, CD11b^+^ MDSCs and cleaved caspase-3^+^ cancer cells expressed in the vicinity of PDAC by counting cells across three fields of view at ×400 magnification. The average value was calculated after counting across three fields of view.

### Statistical analysis

All statistical analyses were conducted using JMP® Pro, version 15.0.0 (SAS Institute Inc.). Cumulative rates were calculated using the Kaplan–Meier method, and the significance of differences in survival rates was assessed using the log-rank test. Data are presented as median values. Survival data were evaluated using both univariate and multivariate Cox proportional hazards regression analyses. Statistically significant differences were determined using Welch’s *t* test, chi-square test, and the Mann–Whitney–Wilcoxon test. Statistical significance was set at *p* < 0.05. Differences marked with asterisks (*) were considered significant. Each experiment was performed independently at least thrice.

## Results

### High C5a-C5aR1 cytoplasmic axis in tumour cells is associated with poor prognosis of patients with PDAC

**Fig. 1:**
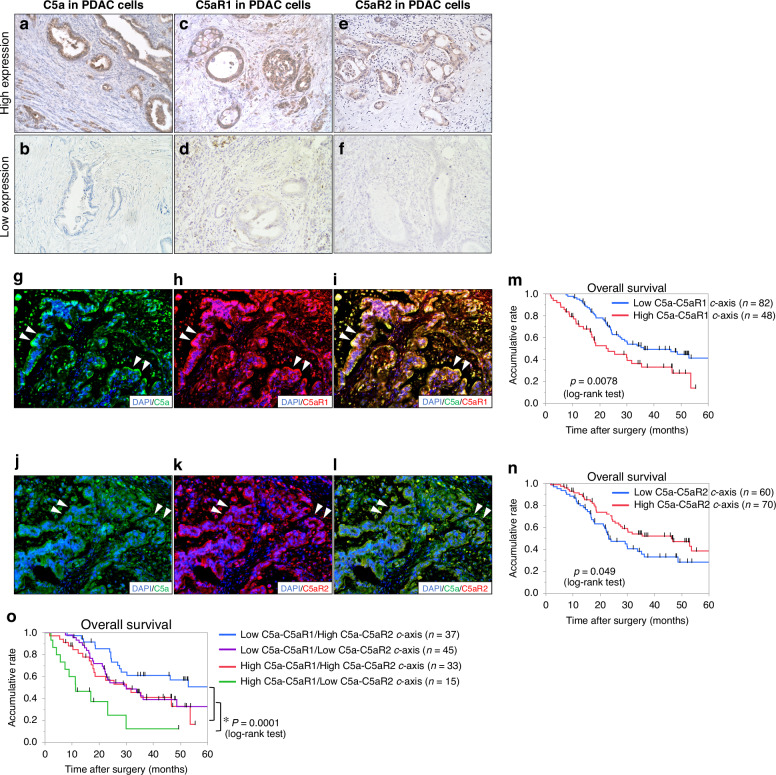
Immunohistochemistry of C5a, C5aR1, and C5aR2 expression in resected human PDAC tissues. **a** High C5a expression in human PDAC cell cytoplasm (*n* = 90). Original magnification: ×200. **b** Low C5a expression in human PDAC cell cytoplasm (*n* = 40). Original magnification: ×200. **c** High C5aR1 expression in human PDAC cell cytoplasm (*n* = 62). Original magnification: ×200. **d** Low C5aR1 expression in human PDAC cell cytoplasm (*n* = 68). Original magnification: ×200. **e** High C5aR2 expression in human PDAC cell cytoplasm (*n* = 102). Original magnification: ×200. **f** Low C5aR2 expression in human PDAC cell cytoplasm (*n* = 28). Original magnification: ×200. **g**–**i** Images of the immunofluorescence (IF) staining of DAPI (blue), C5a (green), and C5aR1 (red) expression in the human PDAC cytoplasm. C5a and C5aR1 are expressed in the PDAC cell cytoplasm, and margin sites were observed (arrowheads). Original magnification: ×200. **j**–**l** Images of IF staining of DAPI (blue), C5a (green), and C5aR2 (red) expression in the human PDAC cytoplasm. C5a and C5aR2 are expressed in the PDAC cell cytoplasm, and margin sites were observed (arrowheads). Original magnification: ×200. **m** Kaplan–Meier analyses for OS of PDAC patients based on PDAC cell cytoplasmic C5a-C5aR1 axis (C5a-C5aR1 c-axis). The high C5a-C5aR1 c-axis group presented significantly shorter OS (*p* = 0.0078, log-rank test) than low C5a-C5aR1 c-axis group. **n** Kaplan–Meier analyses for OS of PDAC patients based on PDAC cell cytoplasmic C5a-C5aR2 axis (defined: C5a-C5aR2 c-axis). The Low C5a-C5aR2 c-axis group presented significantly shorter OS (*p* = 0.049, log-rank test) than the High C5a-C5aR2 c-axis group. **o** Kaplan–Meier analyses for OS of PDAC patients based on PDAC cell cytoplasmic C5a-C5aR1 c-axis and C5a-C5aR2 c-axis combination. The high C5a-C5aR1 c-axis and low C5aR2 c-axis group presented significantly shorter OS than other groups (*p* = 0.0001, log-rank test).

To investigate the expression of C5a, C5aR1, and C5aR2 and their correlation with clinical outcomes, IHC for C5a, C5aR1, and C5aR2 was performed on 130 resected PDAC tissues. C5a and C5aR1 expression was observed in the cytoplasm of tumour cells, as well as in the stroma surrounding the PDAC cells (Fig. 1a–d). In contrast, C5aR2 expression was localised to the cytoplasm of tumour cells (Fig. 1e, f). To validate the specificity of C5a antibody for IHC, we performed IHC for C5/C5b expression to compare the respect expression pattern in PDAC tissues. C5/C5b expression was mainly localised to the stroma surrounding the PDAC cells. The differential staining patterns were observed between C5a and C5/C5b expression in the serial sections of PDAC tissues (Fig. S1A–H). IF staining revealed overlapping expression of C5a/C5aR1 and C5a/C5aR2 in the cytoplasm of PDAC cells (Fig. 1g–l). The cytoplasmic expression levels of C5a, C5aR1, and C5aR2 were divided into two (High/Low) groups; high expression was defined in 90 cases (69.2%) for C5a, 62 cases (47.7%) for C5aR1, and 102 cases (78.5%) for C5aR2, respectively. Among these, 48 cases (36.9%) exhibited high expression of both C5a and C5aR1 in the cytoplasm of PDAC cells, classifying them as a High C5a-C5aR1 cytoplasmic (c-) axis. Similarly, 70 cases (53.8%) were identified as having a High C5a-C5aR2 c-axis due to the high expression of both C5a and C5aR2 in the cytoplasm of PDAC cells. Comparative analysis of clinicopathological parameters between the High C5a-C5aR1 c-axis and Low C5a-C5aR1 c-axis groups indicated a significantly increased tumour volume in the C5a-C5aR1 c-axis group (Table S1).

Kaplan–Meier analysis demonstrated significantly shorter overall survival (OS) (*p* = 0.0078) (Fig. 1m) and disease-free survival (DFS) (Fig. S1I) in the High C5a-C5aR1 c-axis group. No significant differences in the clinicopathological parameters were identified when comparing the two groups with respect to the High C5a-C5aR2 c-axis (Table S2). Conversely, Kaplan–Meier analysis indicated significantly longer OS (*p *= 0.049) (Fig. 1n) and DFS (Fig. S1J) in the group exhibiting a High C5a-C5aR2 c-axis.

The patients were categorised into four groups based on High or Low C5a-C5aR1 and C5a-C5aR2 c-axes. In the group exhibiting a High C5a-C5aR1 c-axis with a Low C5a-C5aR2 c-axis, OS and DFS were significantly shorter than in all other groups (Figs. 1o and S1K). These findings suggest that a strong C5a-C5aR1 c-axis is associated with poor prognosis, whereas a strong C5a-C5aR2 c-axis may contribute to prolonged survival.

### Inhibition of the C5a-C5aR1 axis formation suppresses proliferation in PDAC cells

**Fig. 2:**
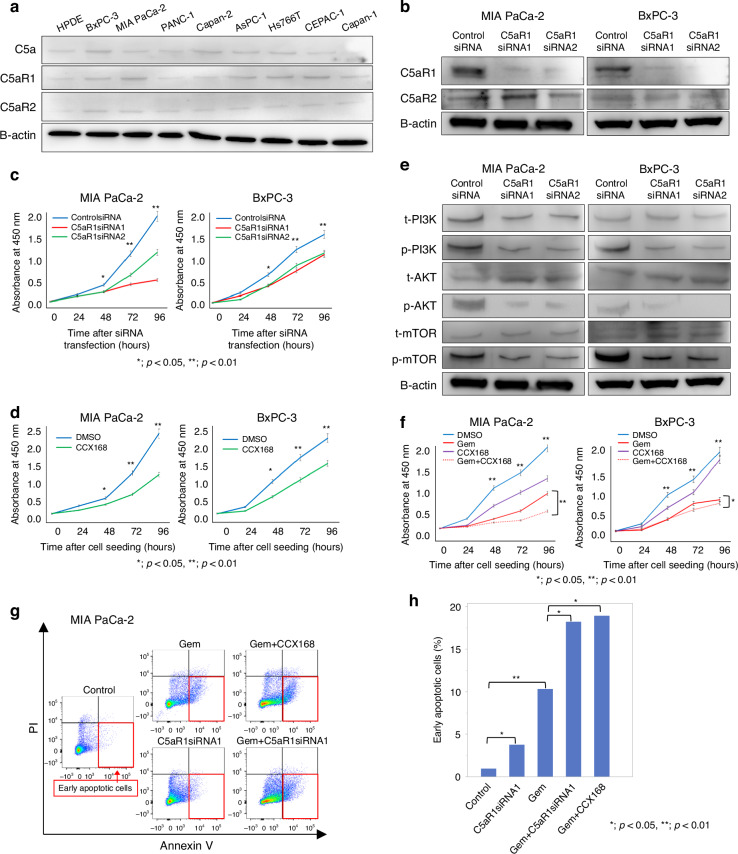
Expression of C5aR1 and the function of C5aR1 in human PDAC cell lines. **a** Western blot analysis of C5a and C5aR1, C5aR2 expression in various human pancreatic cell lines, including cell lines of primary PDAC (BxPC-3, MIA PaCa-2, PANC-1, Capan-2), metastatic PDAC ascites (AsPC-1), lymph node metastasis (Hs766T), and PDAC liver metastases (CFPAC-1, Capan-1). **b** Western blot analysis of the C5aR1 knockdown with C5aR1siRNA transfection in MIA PaCa-2, BxPC-3 cells. The expression of C5aR1 in C5aR1siRNA1, 2-transfected cells was decreased by normalisation with β-actin compared to the control siRNA cells. **c** Proliferation assays using C5aR1siRNAs-transfected cells. The absorbance of MIA PaCa-2 and BxPC-3 cells, which had undergone C5aR1 knockdown with C5aR1siRNA1, 2 transfection, showed significant reduction after 48 h in both cells when compared to the control siRNA group (*p < 0.05, **p < 0.01, Mann–Whitney–Wilcoxon test). **d** The efficacy of C5aR1 selective inhibitor (CCX168) in proliferation assays. The absorbance of MIA PaCa-2 and BxPC-3 cells, which had been seeded with CCX168, showed significant reduction after 48 h in both cells when compared to the control group (*p < 0.05, **p < 0.01, Mann–Whitney–Wilcoxon test). **e** Western blot analysis with C5aR1siRNA-transfected cells (MIA PaCa-2, BxPC-3) showed decreasing expression of p-PI3K, p-AKT, and p-mTOR by normalisation with β-actin compared to the control cells, whereas the expression of t-PI3K, t-AKT, and t-mTOR showed no difference compared to the control cells. **f** The analyses of proliferation using Gemcitabine (Gem) and CCX168. The absorbance of MIA PaCa-2 and BxPC-3 cells, which had been seeded with CCX168, showed significant reduction after 48 h in both cells when compared to the control group (*p < 0.05, **p < 0.001, Mann–Whitney–Wilcoxon test). In addition, proliferation in MIA PaCa-2 cells was significantly inhibited after 48 h in the Gem and CCX168 combined group compared to the Gem or CCX168 group. **g**, **h** Apoptosis assay utilising annexin V and PI using MIA PaCa-2 cells. The number of early apoptotic cells that were positive for Annexin V, but negative for PI, was significantly increased in the group treated with Gem in combination with C5aR1siRNA transfection or CCX168 group compared to Gem group (*p < 0.05, **p < 0.001, Mann–Whitney–Wilcoxon test).

Given the potential involvement of the C5a-C5aR1 c-axis in prognostic deterioration, we conducted in vitro functional experiments on C5aR1 in PDAC cells. We first confirmed the protein expression of C5a, C5aR1, and C5aR2 in HPDE cells and eight human PDAC cell lines using western blotting (Figs. 2a and S2A). Among these cell lines, MIA PaCa-2 and BxPC-3 cells exhibited high C5aR1 expression levels, and subsequent assays were performed using these two cell lines. To validate C5a production in both PDAC cells, we assessed and confirmed mRNA expression of C5, a precursor of C5a (Fig. S2B). To examine whether C5a can act intracellularly and/or extracellularly, we measured C5a protein level in the lysate and supernatant of these cell lines by ELISA. We observed C5a expression in both samples in these two cell lines, implying that C5a functions intracellularly and extracellularly in PDAC (Fig. S2C). By the transfection of C5aR1siRNAs, knockdown of C5aR1 expression was observed, whereas C5aR2 expression did not change in either MIA PaCa-2 or BxPC-3 cells (Figs. 2b and S2D). A significant reduction in proliferation was observed in C5aR1siRNAs transfected cells compared to control cells in both PDAC cell lines (Fig. 2c), indicating that suppression of the C5a-C5aR1 axis could impair PDAC cell proliferation.

CCX168 is an orally administered small-molecule C5aR1 antagonist that selectively inhibits binding C5a to C5aR1 [14–16]. Next, we investigated the inhibitory effects of CCX168 on PDAC cell proliferation. A significant reduction in proliferation was observed in both PDAC cell lines treated with CCX168 compared to control cells (Fig. 2d). We performed western blotting for epithelial-to-mesenchymal transition markers to evaluate their involvement. However, no significant changes in expression were observed in MIA PaCa-2 or BxPC-3 cells following the knockdown of C5aR1 (Fig. S2E).

### Inhibition of C5a-C5aR1 axis suppresses proliferation and enhances chemo-sensitivity through the PI3K/AKT/mTOR signalling pathway

The binding of C5a to C5aR1 initiates endogenous intracellular signalling within the receptor, activates G proteins, and significantly elevates the expression of phosphorylated (p-) PI3K, AKT, and mTOR (p-PI3K/p-AKT/p-mTOR), which promote cell growth and immune cell activation [17–19]. We have previously reported that these proteins are involved in the mechanisms by which PDAC cells acquire resistance to gemcitabine (Gem) treatment through the PI3K/AKT/mTOR signalling pathway [20]. We observed a reduction in the expression of p-PI3K, p-AKT, and p-mTOR in both MIA PaCa-2 and BxPC-3 C5aR1 knockdown cells (Figs. 2e and S2F). Based on these findings, we conducted a cytotoxicity assay using a combination of Gem and CCX168. After 48 h, single treatment with Gem or CCX168 significantly inhibited PDAC cell growth. Notably, in MIA PaCa-2 cells characterised by moderate resistance to Gem [21], the combination of Gem and CCX168 resulted in significantly greater inhibition of proliferation compared to cells treated with Gem (Fig. 2f).

To explore the reason for the enhanced Gem sensitivity induced by the combination of CCX168 in MIA PaCa-2 cells, we conducted an apoptosis assay by flow cytometry with quantified early apoptotic cells. The C5aR1siRNA transfected cells exposed to Gem or the cells treated with a combination of Gem and CCX168 demonstrated a significantly higher rate of early apoptosis compared to the cells treated with Gem alone (Fig. 2g, h). These results suggest that knockdown or inhibition of C5aR1 enhances Gem sensitivity by increasing the apoptosis of PDAC cells.

### The High C5a-C5aR1 stromal axis positively correlates with immunosuppressive feature in the TME of PDAC

**Fig. 3:**
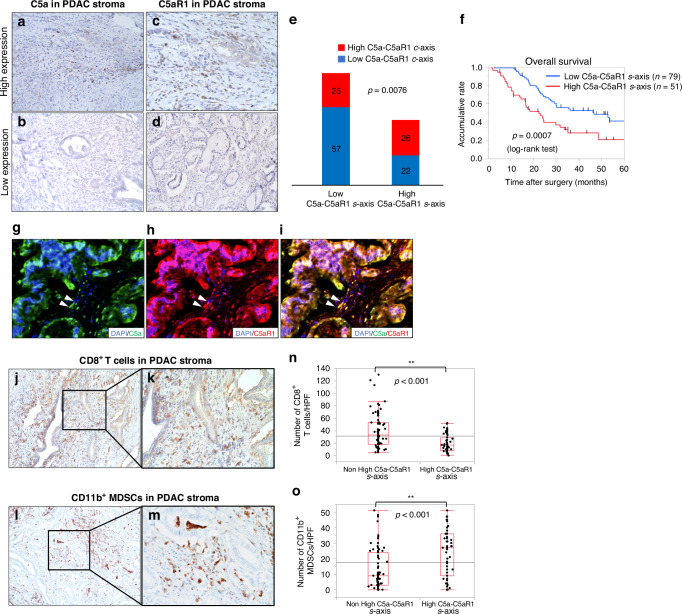
C5a, C5aR1, CD8^+^ T cells, and CD11b^+^ MDSCs in the resected human PDAC stroma. **a** High C5a expression in human PDAC stroma (*n* = 92). Original magnification: ×200. **b** Low C5a expression in human PDAC stroma (*n* = 38). Original magnification: ×200. **c** High C5aR1 expression in human PDAC stroma (*n* = 65). Original magnification: ×200. **d** Low C5aR1 expression in human PDAC stroma (*n* = 65). Original magnification: ×200. **e** In the Low C5a-C5aR1 s-axis group, the proportion of the Low C5a-C5aR1 c-axis was significantly higher than in the High C5a-C5aR1 s-axis group (*p* = 0.0076; Welch’s *t* test). **f** Kaplan–Meier analyses of the OS of patients with PDAC based on the C5a-C5aR1 stromal axis (C5a-C5aR1 s-axis). The C5a-C5aR1 s-axis group had a significantly shorter OS than the Low C5a-C5aR1 s-axis group (*p* = 0.0007, log-rank test). **g**–**i** Images of IF staining of DAPI (blue), C5a (green), and C5aR1 (red) expression in human PDAC stroma. C5a and C5aR1 were expressed in the PDAC stroma and margin sites were observed (arrowheads). Original magnification: ×200. **j**, **k** IHC for CD8 presenting CD8^+^ cytotoxic T cells in the PDAC stoma. Original magnification: ×40, ×400. **l**, **m** IHC for CD11b presenting MDSCs in PDAC stomas. Original magnification: ×40, ×400. **n** The number of CD8^+^ T cells was significantly lower in the C5a-C5aR1 s-axis than in the Low C5a-C5aR1 s-axis (**p < 0.01, Mann–Whitney–Wilcoxon test). **o** The number of CD11b^+^ MDSCs was significantly higher in the C5a-C5aR1 s-axis than in the Low C5a-C5aR1 s-axis (**p < 0.01, Mann–Whitney–Wilcoxon test).

As shown in Fig. 1, IHC for C5a and C5aR1 revealed their expression within the stroma and cytoplasm of tumour cells in PDAC tissues. We evaluated high stromal expression of C5a and C5aR1 in 92 (70.8%) and 59 (45.4%) patients, respectively (Fig. 3a–d). Among these, 51 cases (39.2%) exhibited the C5a-C5aR1 stromal (s-) axis, characterised by high expression of both C5a and C5aR1 in the TME of PDAC. IHC revealed a positive correlation between the expression of the C5a-C5aR1 c-axis and the C5a-C5aR1 s-axis in PDAC tissues (p = 0.0076) (Fig. 3e). Kaplan–Meier analyses demonstrated significantly shorter OS (p = 0.0007) (Fig. 3f) and DFS (Fig. S3) in the High C5a-C5aR1 S-axis group. Univariate analysis of OS revealed significant associations with several factors, including high CA19-9 levels, regional lymph node metastases, high tumour volume, High C5a-C5aR1 c-axis, and High C5a-C5aR1 s-axis. In multivariate analysis, regional lymph node metastases, high tumour volume, High C5a-C5aR1 c-axis, and High C5a-C5aR1 s-axis were identified as independent poor prognostic factors (Table 1).

**Table 1:** Univariate and multivariate analyses for overall survival in PDAC patients.

	Total (*n* = 130)	Univariate analysis		Multivariate analysis	
		Hazard ratio (95% CI)	*p* value	Hazard ratio (95% CI)	*p* value
Age (years) (65</≦65)	100/30	1.50 (0.83–2.70)	0.17		
Gender (male/female)	64/66	1.22 (0.76–1.94)	0.41		
CA19-9 (500≦/<500 U/ml)	96/34	2.06 (1.15–3.69)	0.015*	1.70 (0.96–3.01)	0.07
Regional lymph node metastasis (+/−)	91/39	2.21 (1.17–4.19)	0.015*	2.24 (1.22–4.10)	0.009*
Tumour volume (≥6283, <6283 mm^3^)	71/59	3.60 (2.10–6.17)	<0.0001*	2.31 (1.68–5.52)	0.0002*
C5a-C5aR1 c-axis (High/Low)	48/82	1.89 (1.17–3.04)	0.009*	1.77 (1.02–3.07)	0.04*
C5a-C5aR2 c-axis (High/Low)	70/60	0.62 (0.39–1.00)	0.050	0.65 (0.91–2.57)	0.11
C5a-C5aR1 s-axis (High/Low)	51/79	2.22 (1.38–3.57)	0.001*	2.55 (1.51–4.30)	0.0005*

Cox proportional hazard model, *: significant value.

To assess the correlation between the C5a-C5aR1 s-axis and the immune TME, CD8^+^ T cells, which exhibit antitumour immunoactivities, and CD11b^+^ MDSCs, which are involved in immunosuppression and tumour development, were quantified by IHC (Fig. 3j–m). CD8^+^ T cells were significantly decreased in the stroma surrounding PDAC tissues where the C5a-C5aR1 s-axis was observed (Fig. 3n). In contrast, CD11b^+^ MDSCs were significantly increased in the High C5a-C5aR1 s-axis group compared to those in the non-forming group (Fig. 3o). The High C5a-C5aR1 S-axis was associated with a decrease in cytotoxic T cells and an increase in MDSCs, suggesting that the subpopulation exhibited an immunosuppressive TME of PDAC.

### Suppression of the C5a-C5aR1 axis decreases cell proliferation and improves gemcitabine-resistance in murine PDAC cells

**Fig. 4:**
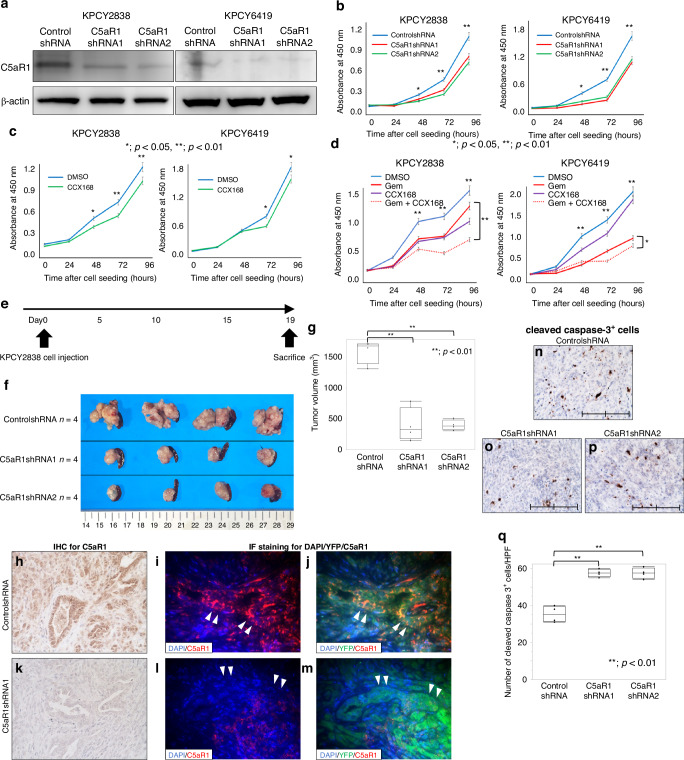
Expression of C5aR1 and the function of C5aR1 in murine PDAC cells and orthotopic transplantation mouse model using shRNA-transfected KPCY cells. **a** Western blot analysis of C5aR1 knockdown with C5aR1shRNA transfection in KPCY2838, KPCY6419 cells. The expression of C5aR1 in C5aR1shRNA1, 2-transfected cells was decreased by normalisation with β-actin compared to the control cells. **b** Proliferation assay analysis compared with C5aR1shRNA-transfected cell lines. The absorbance of KPCY2838 and KPCY6419 cells, which had undergone C5aR1 knockdown with C5aR1shRNA1, 2 transfection, showed significant reduction after 48 h in both cells when compared to the control group (*p < 0.05, **p < 0.01, Mann–Whitney–Wilcoxon test). **c** Proliferation assay analysis compared with CCX168. The absorbance of KPCY2838 and KPCY6419 cells seeded with CCX168 showed significant reduction after 48 h in KPCY2838 cells; 72 h in KPCY6419 when compared to control group (*p < 0.05, **p < 0.01, Mann–Whitney–Wilcoxon test). **d** Proliferation assay analysis compared with Gem and CCX168. Proliferation in KPCY2838 and KPCY6419 cells exposed with CCX168 showed a significant reduction after 48 h in both cells when compared to control group (*p < 0.05, **p < 0.01, Mann–Whitney–Wilcoxon test). Proliferation in KPCY2838 cells was significantly inhibited after 48 h in the Gem and CCX168 combined group compared to the Gem or CCX168 group. **e** Experimental design of the in vivo experiment using KPCY2838 cells, which were transfected using two C5aR1shRNAs. **f** Images of tumours removed after day 19 from orthotopic transplantation using KPCY2838 cells. **g** The tumour volume was significantly reduced in the C5aR1shRNA 1, 2 groups compared to the control group (**p < 0.01, Mann–Whitney–Wilcoxon test). **h**–**m** Images of IHC for C5aR1 in tumour tissues. Compared to the control group, we confirmed that C5aR1 knockdown resulted in a reduction in the expression of C5aR1 in the C5aR1shRNA group. Images of IF staining for DAPI (blue), YFP (green), and C5aR1 (red) expression in tumour tissues. Original magnification: ×200. **n**–**p** Images of IHC for cleaved caspase-3^+^ cells. Original magnification: ×400. **q** The number of cleaved caspase-3^+^ cancer cells was significantly higher in the two C5aR1shRNA groups than in the controlshRNA group (**p < 0.01, Mann–Whitney–Wilcoxon test).

Using two murine PDAC cells, KPCY2838 and KPCY6419, in vitro analyses were conducted to establish an in vivo orthotopic transplantation mouse model. The expression of C5aR1 in these two murine PDAC cell lines and knockdown of C5aR1 expression by transfected with C5aR1shRNAs were verified by western blotting (Fig. 4a). Compared to the control shRNA-treated cells, the proliferation of murine PDAC cells treated with C5aR1shRNAs was significantly suppressed (Fig. 4b). Furthermore, cell cytotoxicity assays were performed using KPCY2838 and KPCY6419 cells treated with CCX168, and a significant reduction in proliferation was observed in both KPCY cell lines treated with CCX168 compared to the control cells (Fig. 4c). Moreover, cell proliferation was significantly reduced in the combination group treated with Gem and CCX168 compared with that in the Gem group (Fig. 4d). These results described that inhibition of the C5a-C5aR1 axis decreases cell proliferation and improves Gem cytotoxicity in murine PDAC cells as well as human PDAC cells.

### Suppression of C5aR1 decreases tumour volume in orthotopic transplantation model

Orthotopic transplantation was performed using mouse PDAC cells treated with two C5aR1shRNAs and Control shRNA (Fig. 4e). KPCY2838 cells transfected with control or C5aR1shRNAs were inoculated into the tail of the pancreas. Upon sacrifice, the tumour volume was significantly smaller in the C5aR1shRNA-treated group compared than in the control group (Fig. 4f, g). IHC and IF staining for C5aR1 confirmed low expression in the tumour cells of the tissue from the C5aR1shRNA-treated group (Fig. 4h–m), and the number of cleaved caspase-3^+^ apoptotic cancer cells was significantly higher in the C5aR1shRNA-treated group than in the control group (Fig. 4n–q), thereby validating in vivo functional regulation of C5aR1 effectively suppressed tumour growth. These results indicate that C5aR1 knockdown suppressed the increase in tumour volume in vivo.

### Inhibition of the C5a-C5aR1 axis fosters immune antitumour effects on PDAC in vivo

**Fig. 5:**
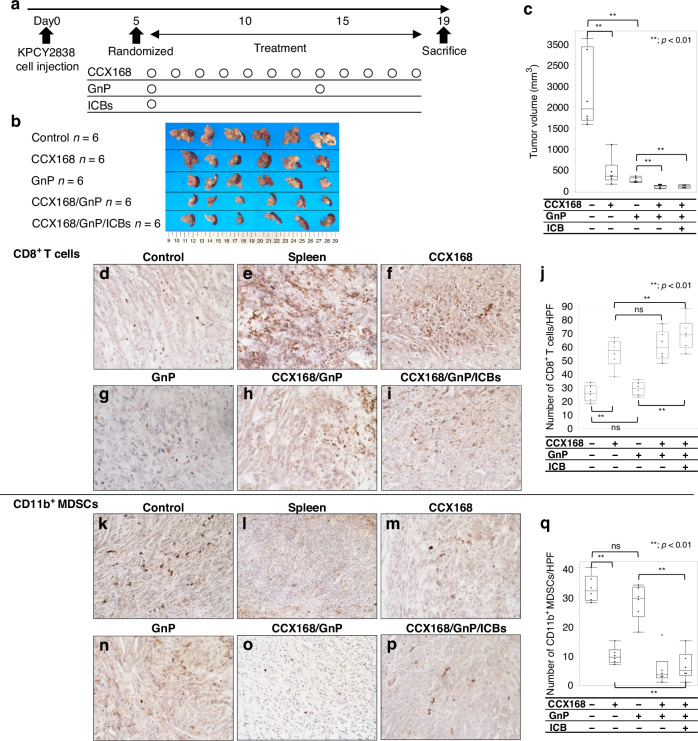
A preclinical study in an orthotopic transplantation mouse model. **a** Experimental design of an in vivo experiment using KPCY2838 cells transfected with C5aR1shRNA. **b** Images of the resected tumours of orthotopic transplantation using KPCY2838 cells. **c** In the CCX168 or GnP groups, the tumour volume was significantly reduced compared to the control group. Furthermore, compared to the CCX168 or GnP groups, a significant reduction in the tumour volume was observed in the CCX168/GnP or CCX168/GnP/immune checkpoint blockades (ICBs) groups (**p < 0.01, Mann–Whitney–Wilcoxon test). **d**–**i** Images of IHC for CD8^+^ T cells. Original magnification: ×400. **j** The number of CD8^+^ T cells was significantly higher in the CCX168 group than in the control or GnP groups. In addition, the CX168/GnP/ICBs group showed a significant increase in cell count compared to the CCX168 or GnP groups (**p < 0.01, Mann–Whitney–Wilcoxon test). **k**–**p** Images of IHC for CD11b^+^ MDSCs. Original magnification: ×400. **q** The number of CD11b^+^ MDSCs was significantly lower in the CCX168 group than in the control or GnP groups. Additionally, the CCX168/GnP/ICBs group showed a significant decrease in the number of CD11b^+^ MDSCs compared to the CCX168 or GnP groups (**p < 0.01, Mann–Whitney–Wilcoxon test).

To investigate the influence of the C5a-C5aR1 axis activation by the C5aR1 antagonist CCX168, orthotopic transplantation was performed in a preclinical study with a five-arm treatment, as shown in the experimental design in Fig. 5a. Tumour volumes in both the CCX168- and GnP-treated groups were significantly decreased compared to those in the untreated group (Fig. 5b, c). Moreover, the combination of CCX168 and GnP (CCX168/GnP group) demonstrated a significantly greater reduction in tumour volume than either CCX168 or GnP alone.

Finally, immunostaining for CD8 and CD11b was performed to quantify the number of CD8^+^ T cells and CD11b^+^ MDSCs in the stroma surrounding PDAC cells. In the GnP group, the number of CD8^+^ T cells was not significantly different from that in the untreated group (Fig. 5d–j). In contrast, the CCX168 group showed a significant increase in CD8^+^ T cells compared to the untreated group. Notably, all three groups in which CCX168 was used had significantly increased numbers of CD8^+^ T cells compared to the GnP group (Fig. 5j). Furthermore, the number of CD8^+^ T cells significantly increased in the combination of CCX168 and ICBs (CCX168/GnP/ICBs group) compared to the CCX168 only group. Contrary to these findings, CCX168 treatment resulted in an obvious decrease in the number of MDSCs compared to the untreated or GnP alone groups (Fig. 5k–q). Additionally, the combination of CCX168 and ICBs demonstrated a significantly impairment of the number of MDSCs compared to CCX168 or GnP alone (Fig. 5q). In terms of the apoptosis of cancer cells, the number of apoptotic cancer cells was significantly higher in the CCX168 or GnP group than in the control group. Interestingly, the CCX168/GnP and CCX168/GnP/ICBs groups showed a significant increase in the apoptotic cancer cells compared to the CCX168 or GnP group (Fig. S5A–F). Taken together, these findings suggest that inhibition of the C5a-C5aR1 axis by CCX168 improves the immunosuppressive TME and the efficacy of chemo-immunotherapy in PDAC.

## Discussion

We investigated the functional roles of the C5a-C5aR1 cytoplasmic and stromal axes as potential targets for PDAC treatment in the TME. The C5a-C5aR1 c- and s-axes correlated with poor outcomes owing to the facilitation of tumour growth and immunosuppressive activity, leading to PDAC progression. Furthermore, C5aR1 knockdown or C5aR1 inhibition suppressed PI3K/AKT/mTOR signal transduction and fostered Gem sensitivity in PDAC cells in vitro. Additionally, the C5aR1 inhibition combined with GnP therapy facilitated tumour regression, and enhanced the antitumour immunity by adding ICB treatment in the immune TME of PDAC in vivo (Fig. S6).

C5a, an anaphylatoxin generated by the cleavage of C5 during complement system activation, primarily exerts its effects by activating C5aR1 and C5aR2 receptors [22]. The impact of C5a on tumour progression in the TME varies depending on the tumour type and the characteristics of the TME [7, 23–26]. Activation of C5aR1 in the cytoplasm triggers downstream signalling pathways mediated by G protein-coupled receptors, such as the PI3K/AKT and NF-κB pathways, which are thought to enhance tumour cell proliferation, invasion, and metastasis [27–29]. Furthermore, Nabizadeh et al. reported a reduction in melanoma tumour growth in C5aR1-deficient mice, but an increase in C5aR2-deficient mice [30]. Consistent with this finding, our study demonstrated that the C5a-C5aR1 c-axis is correlated with poor prognosis, whereas the C5a-C5aR2 c-axis is correlated with favourable outcomes in PDAC tissues. There may an association with the activity of C5aR2 as a negative modulator of C5aR1 expression.

The C5a-C5aR1 s-axis adversely affects tumour immunity by mobilising MDSCs and enhancing CD8^+^ T cell suppressive capability. Markiewski demonstrated that the C5a/C5aR1 axis is associated with the activation, recruitment, and distribution of MDSCs in tumours [31]. Our study revealed that high expression of the C5a-C5aR1 c- and s-axes affects the immune TME of PDAC, increasing MDSCs and reducing CD8^+^ T cells, thereby promoting immune evasion and suppressing antitumour responses.

Advances in understanding the complement pathway and the development of biological and small-molecule drugs have led to the development of therapies that directly target this system. The C5aR1 inhibitor functions in both intracellular and extracellular PDAC, as C5a itself is a peptide that exerts its effects by binding to C5aR1 on the surface of target cells such as cancer cells and immune cells [32–34]. CCX168, a selective C5aR1 inhibitor for ANCA-associated vasculitis, has demonstrated clinical efficacy [16], although its potential antitumour effects in oncology are yet to be explored [17–19]. In preclinical models of lung cancer, Ajona demonstrated that a combination of C5a blockade and ICB significantly reduced the proportion of tumour-infiltrating MDSCs and attenuated local tumour growth and metastatic spread [28]. Very recently, it is reported that the combination of CCX168 and anti-PD-1 antibody improved their antitumour activity and attenuated the growth of murine anaplastic thyroid cancer cell-derived tumours in a murine model [32]. Our findings indicate that CCX168 can enhance the efficacy of Gem-based therapy and increase antitumour immunity by adding ICBs, through the accumulation of cytotoxic T cells and attenuation of the MDSCs population in the PDAC stroma. In this study, it is possible that the number of MDSCs was low enough that no obvious difference was observed between the CCX168/GnP group and the CCX168/GnP/ICBs group.

This study had some inherent limitations. We did not examine intracellular C5aR2 functions and whether there is a reciprocal relationship between C5aR1 and C5aR2 in PDAC cells. Further, the exact mechanism involved in the suppressive effect of the C5a-C5aR1 s-axis on CD8^+^ T cell infiltration in the immune TME of PDAC needs to be comprehensively analysed. In future research, it will be critical to investigate the downstream signalling pathways involved in C5a-C5aR1 interactions to clarify the mechanisms associated with tumour growth in the immune TME of PDAC.

In conclusion, our findings suggested that the C5a-C5aR1 axis may exert a tumour-promoting effect on the immune TME of PDAC. Administration of a C5aR1 inhibitor appears to modulate antitumour immunity within both tumour cells and the surrounding stroma, thereby warranting future clinical applications. To facilitate the clinical use of complement immunomodulation therapy, it is essential to investigate the specific mechanisms underlying the C5a-C5aR1 axis in the immune TME of PDAC.

## Supplementary information

Supplementary Figures S1-6

Supplementary Table S1

Supplementary Table S2

Supplementary legends and antibody lists

## Data Availability

The datasets used and/or analysed during the current study are available from the corresponding author on reasonable request.
