# Supplementary material for: Inhibition of C5a-C5aR1 axis suppresses tumour progression by enhancing antitumour immunity and chemotherapeutic effect in pancreatic ductal adenocarcinoma
Source: Br J Cancer. 2025 Oct 3;133(12):1791–801. doi: 10.1038/s41416-025-03185-0 (PMC12690149; doi:10.1038/s41416-025-03185-0)
Supplement: Supplementary file 2 — Supplementary Table S1 [file 41416_2025_3185_MOESM2_ESM.docx]

Supplementary Table S1: Characteristics of PDAC patients in IHC analysis for expression of C5a-C5aR1 c-axis

|  | High C5a-C5aR1 c-axis (n=48) | Low C5a-C5aR1 c-axis (n=82) | P value |
| --- | --- | --- | --- |
| Age (y.o. median: range) | 71 (38-88) | 70 (54-87) | 0.43 |
| Sex (male/female) | 28/20 | 36/46 | 0.15 |
| BMI (kg/m^2^, median: range) | 22.3 (14.4-28.5) | 21.6 (16.8-31.1) | 0.23 |
| CA19-9 (U/ml, median: range) | 104.0 (0.1-5590) | 146.9 (0.1-8220) | 0.39 |
| Histological grade (wel, mod/por, others) | 36/12 | 73/9 | 0.18 |
| Tumor volume (mm^3^, ≥6283 / 6283>) | 34/14 | 37/45 | 0.006* |
| pT stage (pT3,4/pT1,2) | 6/42 | 8/74 | 0.52 |
| Regional lymph node metastasis (+/−) | 37/11 | 54/28 | 0.23 |
| Lymphatic invasion (+/−) | 44/4 | 77/5 | 0.96 |
| Venous invasion (+/−) | 40/8 | 73/9 | 0.89 |
| Perineural invasion (+/−) | 43/5 | 77/5 | 0.88 |
| Hematogenous recurrence (+/−) | 13/35 | 23/59 | 0.62 |
| Local recurrence (+/−) | 15/33 | 24/58 | 0.81 |

^*^: significant value
