# Supplementary material for: Inhibition of C5a-C5aR1 axis suppresses tumour progression by enhancing antitumour immunity and chemotherapeutic effect in pancreatic ductal adenocarcinoma
Source: Br J Cancer. 2025 Oct 3;133(12):1791–801. doi: 10.1038/s41416-025-03185-0 (PMC12690149; doi:10.1038/s41416-025-03185-0)
Supplement: Supplementary file 3 — Supplementary Table S2 [file 41416_2025_3185_MOESM3_ESM.docx]

Supplementary Table S2: Characteristics of PDAC patients in IHC analysis for expression of C5a-C5aR2 c-axis

|  | High C5a-C5aR2 c-axis (n=70) | Low C5a-C5aR2 c-axis (n=60) | P value |
| --- | --- | --- | --- |
| Age (y.o. median: range) | 70 (41-87) | 73 (38-88) | 0.09 |
| Sex (male/female) | 36/34 | 28/32 | 0.60 |
| BMI (kg/m^2^, median: range) | 21.8 (14.4-27.8) | 21.8 (16.8-31.1) | 0.88 |
| CA19-9 (U/ml, median: range) | 139.0 (0.1-5590) | 99.2 (0.1-8220) | 0.59 |
| Histological grade (wel, mod/ por, others) | 58/12 | 51/9 | 0.51 |
| Tumor volume (mm^3^, median) | 6283 (377-30140) | 6957 (586-50265) | 0.10 |
| pT stage (pT3,4/pT1,2) | 5/65 | 9/51 | 0.16 |
| Regional lymph node metastasis (+/−) | 48/22 | 43/17 | 0.58 |
| Lymphatic invasion (+/−) | 66/4 | 55/5 | 0.44 |
| Venous invasion (+/−) | 59/11 | 54/6 | 0.45 |
| Perineural invasion (+/−) | 65/5 | 55/5 | 0.35 |
| Hematogenous recurrence (+/−) | 22/48 | 14/46 | 0.33 |
| Local recurrence (+/−) | 19/51 | 20/40 | 0.45 |
