# Supplementary material for: Inhibition of C5a-C5aR1 axis suppresses tumour progression by enhancing antitumour immunity and chemotherapeutic effect in pancreatic ductal adenocarcinoma
Source: Br J Cancer. 2025 Oct 3;133(12):1791–801. doi: 10.1038/s41416-025-03185-0 (PMC12690149; doi:10.1038/s41416-025-03185-0)
Supplement: Supplementary file 4 — Supplementary legends and antibody lists [file 41416_2025_3185_MOESM4_ESM.docx]

**Supplementary legends and antibody lists**

**Supplementary Fig. S1.** Immunohistochemistry of C5a and C5/C5b expression in resected human PDAC tissues. The differential staining patterns between C5a and C5/C5b expression in the serial sections of PDAC tissues. (A-F) C5a expression was observed in the cytoplasm of tumor cells, as well as in the stroma surrounding the PDAC cells whereas C5/C5b expression was not observed in the cytoplasm of tumor cells but in the stroma surrounding the PDAC cells (arrowheads). (G-H) IHC for C5a and C5/C5b in normal liver tissues (positive control). Original magnification: ×200. Disease-free survival analysis. (I) Kaplan–Meier analysis of disease-free survival (DFS) of patients with PDAC based on the C5a-C5aR1 c-axis. The High C5a-C5aR1 c-axis group presented a significantly shorter DFS (p=0.034, log-rank test) than the Low C5a-C5aR1 c-axis group. (J) Kaplan–Meier analyses of DFS in patients with PDAC based on the C5a-C5aR2 c-axis. The Low C5a-C5aR2 c-axis group presented a significantly shorter DFS (p=0.043, log-rank test) than the High C5a-C5aR2 c-axis group. (K) Kaplan–Meier analyses of OS of patients with PDAC based on the PDAC cell cytoplasmic C5a-C5aR1 and C5a-C5aR2 c-axis combinations. The high C5a-C5aR1 c-axis and low C5aR2 c-axis group presented significantly shorter DFS than the other groups (p=0022, log-rank test).

**Supplementary Fig. S2.** (A) Whole images of the uncut gels for C5a, C5aR1, and C5aR2 by western blotting. (B) C5 mRNA expression by quantitative RT-PCR in MIA PaCa-2, BxPC-3 cells. (C) C5a protein level in the lysate and supernatant of these two PDAC cell lines by ELISA. (D) Western blot analysis of the C5aR1 knockdown with C5aR1siRNA transfection in MIA PaCa-2, BxPC-3 cells. The expression of C5aR1 in C5aR1siRNA1, 2-transfected cells were decreased by normalized with β-actin compared to the control siRNA cells. (E) Western blot analysis with C5aR1siRNA transfected cells (MIA PaCa-2, BxPC-3) showed no difference of the expression of E-cadherin, Vimentin, and Snail normalized with β-actin compared to the control cells. (F) Western blot analysis of C5aR1siRNA transfected cells (MIA PaCa-2, BxPC-3) showed a decrease in the expression of p-PI3K, p-AKT, and p-mTOR compared to control cells, whereas the expression of t-PI3K, t-AKT, and t-mTOR showed no difference compared to control cells.

**Supplementary Fig. S3.** Analysis of DFS. Kaplan–Meier analyses of the DFS of patients with PDAC based on the C5a-C5aR1 s-axis. The High C5a-C5aR1 s-axis group presented a significantly shorter DFS (p<0.001, log-rank test) than the Low C5a-C5aR1 s-axis group.

**Supplementary Fig. S4.** (A) Western blot analysis of C5aR1 knockdown with C5aR1shRNA transfection in KPCY2838, KPCY6419 cells. The expression of C5aR1 in C5aR1shRNA1, 2-transfected cells were decreased by normalized with β-actin compared to the control cells. (B-D) Images of IHC for CD8^+^ T cells. Original magnification: ×400. (E) The number of CD8^+^ T cells was no significant difference in the groups (Mann–Whitney–Wilcoxon test). (F-H) Images of IHC for CD11b^+^ MDSCs. Original magnification: ×400. (i) The number of CD11b^+^ MDSCs was no significant difference in the groups (Mann–Whitney–Wilcoxon test).

**Supplementary Fig, S5.** (A–E) Images of IHC for cleaved caspase-3^+^ cells. Original magnification: ×400. (f) The number of cleaved caspase-3^+^ cancer cells was significantly higher in the CCX168 or GnP group than in the control group. In addition, the CCX168/GnP and CCX168/GnP/ICBs groups showed a significant increase in cell count compared to the CCX168 or GnP group (**p<0.01, Mann–Whitney–Wilcoxon test).

**Supplementary Fig. S6.** Schema of functional roles of the C5a-C5aR1 c-axis/s-axis in the immune TME of PDAC.

**Supplementary Table S1.** Characteristics of patients with PDAC in IHC analysis for C5a-C5aR1 c-axis expression

**Supplementary Table S2.** Characteristics of patients with PDAC in IHC analysis for C5a-C5aR2 c-axis expression

**Antibody list**

・C5aR1 Antibody: cat. no. A1900, ABclonal, Clonality: Polyclonal, Isotype: IgG, Host: Rabbit, IHC・IF Degree of dilution: 1:150, WB Degree of dilution: 1:1000

・C5aR2 Antibody: cat. no. HPA016629, Sigma-Aldrich, Clonality: Polyclonal, Isotype: IgG, Host: Rabbit, IHC・IF Degree of dilution: 1:75, WB Degree of dilution: 1:1000

・C5a Antibody: cat. no. MA5-43875, Thermo Fisher, Clonality: monoclonal, Isotype: IgG, Host: Mouse, IHC・IF Degree of dilution: 1:100, WB Degree of dilution: 1:1000

・C5/C5b Antibody: cat. no. HA255621, ABclonal, Clonality: monoclonal, Isotype: IgG, Host: Rabbit, IHC Degree of dilution: 1:100

・CD8 Antibody: cat. no. ab217344, Abcam, Clonality: polyclonal, Isotype: IgG, Host: Rabbit, IHC Degree of dilution: 1:200

・CD11b Antibody: cat. no. D6X1N, Cell Signaling Technology, Clonality: monoclonal, Isotype: IgG, Host: Rabbit, IHC Degree of dilution: 1:200

・Cleaved caspase-3 Antibody: cat. no. D6X1N, Cell Signaling Technology, Clonality: monoclonal, Isotype: IgG, Host: Rabbit, IHC Degree of dilution: 1:150

・PI3K Kinase Antibody: cat. no. #4292, Cell Signaling Technology, Clonality: polyclonal, Isotype: IgG, Host: Rabbit, WB Degree of dilution: 1:1000

・Phospho-PI3 Kinase Antibody: cat. no. #4228, Cell Signaling Technology, Clonality: polyclonal, Isotype: IgG, Host: Rabbit, WB Degree of dilution: 1:1000

・Akt Antibody: cat. no. #9272, Cell Signaling Technology, Clonality: polyclonal, Isotype: IgG, Host: Rabbit, WB Degree of dilution: 1:1000

・Phospho-Akt Antibody: cat. no. #9271, Cell Signaling Technology, Clonality: polyclonal, Isotype: IgG, Host: Rabbit, WB Degree of dilution: 1:1000

・mTOR Antibody: cat. no. #2983, Cell Signaling Technology, Clonality: polyclonal, Isotype: IgG, Host: Rabbit, WB Degree of dilution: 1:1000

・Phospho-mTOR Antibody: cat. no. #2971, Cell Signaling Technology, Clonality: polyclonal, Isotype: IgG, Host: Rabbit, WB Degree of dilution: 1:1000

・E-cadherin antibody: cat. no. sc-8426, Santa Cruz Biotechnology, Clonality: polyclonal, Isotype: IgG, Host: Rabbit, WB Degree of dilution: 1:1000

・Vimentin antibody: cat. no. #5741, Cell Signaling Technology, Clonality: polyclonal, Isotype: IgG, Host: Rabbit, WB Degree of dilution: 1:1000

・Snail antibody: cat. no.#3879, Cell Signaling Technology, Clonality: polyclonal, Isotype: IgG, Host: Rabbit, WB Degree of dilution: 1:1000
